# Perioperative Management of Single Lung Transplantation for a Chronic Obstructive Lung Disease Patient with Congenital Alpha_2_-Plasmin Inhibitor Deficiency

**DOI:** 10.70352/scrj.cr.25-0230

**Published:** 2025-06-25

**Authors:** So Miyahara, Shohei Mitsumata, Shiro Kaneda, Jun-ichi Wakahara, Ryu-ichi Waseda, Toshihiko Sato, Takeshi Shiraishi

**Affiliations:** Department of General Thoracic, Breast and Pediatric Surgery, Fukuoka University School of Medicine and Hospital, Fukuoka, Japan

**Keywords:** lung transplantation, α_2_-plasmin inhibitor, congenital, surgical management, rotational thromboelastometry

## Abstract

**INTRODUCTION:**

Coagulation disorders can lead to massive perioperative bleeding regardless of the type of surgery. Their preoperative identification is essential (from a complete history of bleeding tendency) and steps should be taken to mitigate such complications at the time of surgery. Alpha_2_-plasmin inhibitor (α2-PI) deficiency is a rare congenital coagulation disorder resulting in activation of fibrinolysis and requiring specific treatment with antifibrinolytic agents. Lung transplantation has not been previously reported in a patient with α2-PI deficiency.

**CASE PRESENTATION:**

A 46-year-old female affected by chronic obstructive pulmonary disease with congenital α2-PI deficiency was referred to our hospital for cadaveric lung transplantation. Due to a previous history of intramedullary hemorrhage, we conducted lung transplantation with prophylactic administration of fresh frozen plasma (FFP) and tranexamic acid during surgery. We used the point of care test (POC) rotational thromboelastometry (ROTEM) to diagnose intraoperative coagulopathy. The postoperative course was uneventful, and she was discharged from the hospital 42 days after lung transplantation. Six months have passed since transplant, and she is still attending outpatient clinics in good health and with no record of bleeding episodes.

**CONCLUSIONS:**

Lung transplantation for a patient with α2-PI deficiency was safely performed with the use of planned FFP transfusion and tranexamic acid. A POC ROTEM testing approach to perioperative management was useful during lung transplantation.

## INTRODUCTION

Alpha_2_-plasmin inhibitor (α2-PI) is a major regulator of fibrinolysis and an essential factor involved in hemostasis.^1)^ Congenital α2-PI deficiency is inherited in an autosomal recessive manner and is rare; delayed bleeding associated with this disease occurs in response to trauma, surgery, or dental procedures.^1,2)^ In this report, we describe the first successful lung transplantation for the treatment of advanced chronic obstructive lung disease with congenital α2-PI deficiency.

## CASE PRESENTATION

**Fig. 1:**
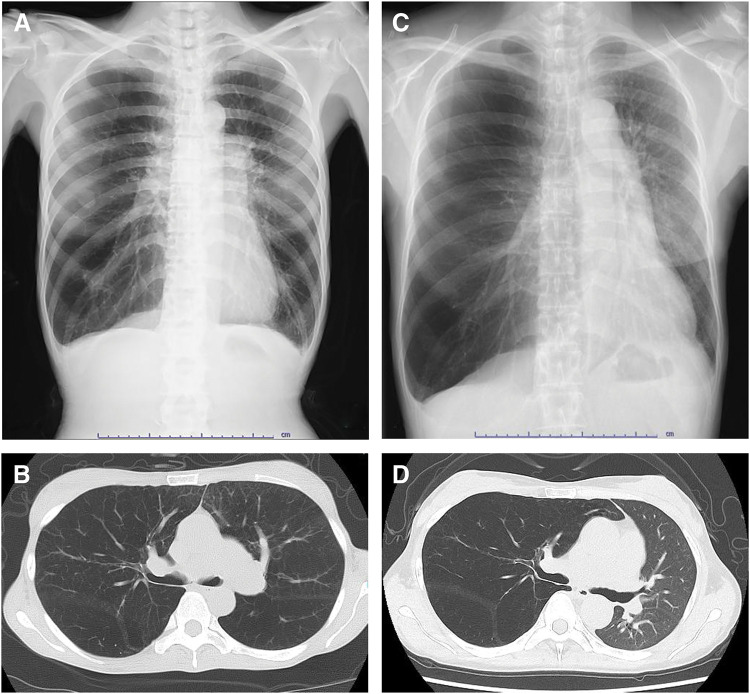
(**A**) Preoperative chest X-ray. (**B**) Preoperative chest computed tomography. (**C**) Chest X-ray during the first 6 months after lung transplantation. (**D**) Computed tomography during the first 6 months after lung transplantation.

A 46-year-old female with chronic obstructive pulmonary disease, diagnosed based on radiological findings (**Fig. 1A** and **1B**), was referred to our hospital for cadaveric lung transplantation due to the gradual deterioration of her respiratory status. She has no previous smoking history. As a child, she had a surgical history of intramedullary hematoma associated with congenital α2-PI deficiency, and her two sisters, but not her two brothers, had also been diagnosed with the same disease. Although she had undergone two previous cesarean births, there was no postoperative bleeding when administered intravenous tranexamic acid. A preoperative blood screening showed a high platelet count (946 × 10^3^/μL, normal 158–348 × 10^3^/μL) and an abnormal prothrombin time (13.1 s, normal 9.8–12.1 s). Fibrinogen (132 mg/dl, normal 200–400 mg/dl) was slightly decreased. The activated partial thromboplastin time (APTT), fibrin/fibrinogen degradation products (FDP), and D-dimer were within normal limits. Although plasmin inhibitor complex (PIC) was also within normal limits, α2-PI activity (32%, normal <85%–115%) was severely decreased. Prior to lung transplantation, a combination of fresh frozen plasma (FFP) transfusion and continuous intravenous tranexamic acid was planned to prevent unexpected bleeding.

**Fig. 2:**
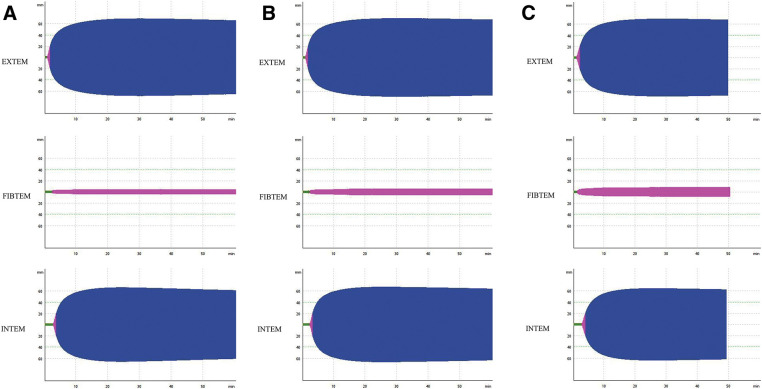
(**A**) Basal (preoperative) values: EXTEM assay, FIBTEM assay, and INTEM assay. (**B**) Four hours after FFP administration: EXTEM assay, FIBTEM assay, and INTEM assay. (**C**) Twelve hours after transplantation: EXTEM assay, FIBTEM assay, and INTEM assay. EXTEM, extrinsic coagulation cascade; FFP, fresh frozen plasma; FIBTEM, fibrinogen on the final clot strength; INTM, intrinsic coagulation cascade

After a wait of 41 months, she underwent cadaveric left lung transplantation. Oral tranexamic acid (1500 mg/day) was administered immediately after admission to our hospital, and four units of FFP was transfused at entry to the operating room. Continuous intravenous tranexamic acid was also given at 2 mg/kg/h during surgery. Point of care test (POC) rotational thromboelastometry (ROTEM) was used during the entire surgical process to monitor coagulation capacity (**Fig. 2A**–**2C**). The recipient underwent a posterolateral incision. After the hilar dissections, bronchial anastomosis was performed with a continuous suture for the membranous part and interrupted suture for the cartilaginous part (4-0 polydioxanone) in an end-to-end fashion. Subsequently, the left atrial anastomosis (5-0 polypropylene) and pulmonary artery end-to-end anastomosis (6-0 polypropylene) were performed in a running fashion. Extracorporeal membrane oxygenation (ECMO) was not required during surgery. Four units of FFP were administered again just before reperfusion. The left lung graft ischemic time was 496 min, and the total operation time was 253 min. Total blood loss was 200 mL. Tranexamic acid was administered intravenously (2 mg/kg/h) until postoperative day 3 (POD 3) and switched to oral administration (1500 mg/day) on POD 4 to POD 14. She was extubated on POD 3 and discharged from the intensive care unit on POD 5 without any complications. She was discharged from the hospital 42 days after lung transplantation. There were no episodes of postoperative bleeding during her hospitalization. The coagulation-fibrinolysis system did not show any significant consistent changes associated with perioperative periods (**Table 1**). The results of a serial chest X-ray and a follow-up chest computed tomography were normal (**Fig. 1C** and **1D**). Respiratory function tests showed an increase in forced expiratory volume in 1s (FEV1) (from preoperative 430 mL to postoperative 1010 mL) and FEV1% (from preoperative 21.6% to postoperative 60.1%), and an improvement in diffusing capacity for carbon monoxide (DLCO) (from preoperative 9.9% to postoperative 6.5%). Meanwhile, postoperative forced vital capacity (FVC) was 1680 mL (preoperative FVC 1990 mL). She remains well 6 months after transplantation without limitations in her daily activities.

**Table 1:** Perioperative blood test

	Preoperative	Postoperative	POD 1	POD 4	POD 15	Normal value
Platelet count, ×10^3^/μL	946	871	508	634	1259	158–348
Prothrombin time, %	70	67	61	85	84	70–130
Activated partial thromboplastin time, s	32.6	29.2	31.0	24.9	23.7	24–34
Fibrinogen, mg/dL	132	147	197	285	272	200–400
FDP, μg/mL	<3	5	7	10	5	≤5
d-Dimer, μg/mL	<0.5	0.8	1.4	8.0	3.4	≤1
PIC, μg/mL	0.1	6.6	7.7	0.3	0.1	<0.8
α2-PI, %	28	42	40	35	39	85–115

FDP, fibrin/fibrinogen degradation products; PIC, plasmin inhibitor-plasmin complex; PI, plasmin inhibit; POD, postoperative day

## DISCUSSION

α2-PI, along with α_1_-antitripsin, is a member of the serine protease inhibitor (serpin) family in plasma. α2-PI functions as a regulator of biological defense systems, mainly serine proteases, such as the blood coagulation-fibrinolysis system and the complement system.^3)^ In many cases, a steady state of neither deficiency nor excess of these factors is physiologically essential, as imbalance can lead to hemorrhagic or thrombotic symptoms. α2-PI is a plasma glycoprotein that inhibits the physiological fibrinolytic process that follows fibrin formation and stabilizes hemostatic plugs. In congenital α2-PI deficiency, these plugs are dissolved prematurely, before injured vessel repair, resulting in a strong hemorrhagic tendency.^1)^

Congenital deficiency of α2-PI is rare and it is inherited in an autosomal recessive manner. Patients with homozygous α2-PI deficiency may exhibit a tendency toward severe bleeding, often presenting in childhood with symptoms similar to those patients with congenital hemophilia. Umbilical bleeding may be the first event of this disease. Furthermore, the unusual symptom of intramedullary hemorrhage into the diaphyses of long bones occurring spontaneously and in response to trauma has been described.^4)^ Other abnormal bleeding, such as prolonged bleeding after lacerations or contusions, gingival bleeding, hemarthroses, subarachnoid and epidural hemorrhage, muscle hematomas, hemothorax, hernia, and severe post adenoidectomy bleeding have been reported. Heterozygous individuals, by contrast, may have milder bleeding or may be asymptomatic in comparison to homozygous ones.^5,6)^ However, symptoms may increase with age as a result of falling plasma levels, causing new symptoms in elderly patients with heterozygous deficiency.^7)^ Although genetic testing was not performed in this case, the possibility of homozygous deletion could not be ruled out considering the history of intramedullary hemorrhage and the sisters’ history of subarachnoid hemorrhage. Therefore, prophylactic administration to reduce intra-operative bleeding was considered essential.

Some anti-fibrinolytic agents including tranexamic acid or α-aminocaproic acid are used to treat patients who bleed, or to avoid hemorrhagic complications in individuals undergoing surgical interventions.^8)^ These agents prevent the binding of plasminogen to fibrin and thereby inhibit endogenous fibrinolysis and stabilize the hemostatic plug or increase the inhibitory activity of α_2_-macroglobulin.^9)^ FFP, which includes α2-PI, can be used as an alternative to anti-fibrinolytic agents. However, due to the rarity of this congenital disorder, optimal prophylactic regimens remain unclear. Furthermore, there is no index of blood examination to assess the real-time activity of α2-PI intraoperatively. Therefore, we decided to address this α2-PI deficiency preoperatively using FFP, and to use continuous intravenous tranexamic acid infusion during and immediately after surgery. Fortunately, the patient was able to be discharged safely without massive bleeding events. In this case, platelets were markedly increased preoperatively, which may have been compensatory for the abnormal fibrinolysis caused by α2-PI deficiency. The administration of FFP and tranexamic acid did not significantly change the laboratory data (**Table 1**), although there may have been no significant problems in the postoperative course of the patient without these treatments. However, as postoperative severe bleeding has been reported in a patient with preoperative normal coagulation tests,^10)^ prophylactic FFP or tranexamic acid should be used.

ROTEM gives a point-of-care evaluation of coagulation function; it measures the viscoelasticity of clots using whole blood throughout the coagulation process and provides a visual assessment of coagulation, from the formation of clots to the accelerated and stabilized dissolution of clots during the fibrinolysis process.^11)^ Because of its simplicity, this test can be performed at the bedside during surgery and experience of its use has been reported in many operations requiring blood transfusion, such as cardiac surgery and liver transplantation.^12,13)^ The advantage of this test is the possibility of separating the activation of the intrinsic coagulation cascade (INTM) and the extrinsic coagulation cascade (EXTEM). In addition, it is possible to monitor the influence of fibrinogen on the final clot strength (FIBTEM). Increased fibrinolytic capacity can also be inferred by attenuation or disappearance of amplitude. The INTEM, EXTEM, and FIBTEM amplitude in this case did not change, indicating no perioperative enhancement of fibrinolysis. This system may also be useful in lung transplant procedures in patients with abnormal coagulation or fibrinolytic function and may minimize the need for blood transfusions.

It is well known that congenital deficiency of α1-antitrypsin, which is a member of the same serpin family as α2-PI, shows no bleeding tendency, but causes emphysema due to excessive neutrophil elastase damage to lung connective tissue.^14)^ On the other hand, an association between α2-PI deficiency and the development of emphysema has not been previously reported. It may be coincidental that this case presented with emphysema; however, the possibility of some causal relationship cannot be ruled out, considering that her sister with α2-PI deficiency presented with, and died from, emphysema.

## CONCLUSIONS

In summary, lung transplantation for a patient with α2-PI deficiency was safely performed with the administration of an FFP transfusion and tranexamic acid.

## DECLARATIONS

### Funding

None.

### Authors’ contributions

S Miy and T Sh drafted the manuscript.

S Mit, JW, SK, RW, and T Sa contributed to patient care and data collection.

All authors critically revised the content and approved the final version.

### Availability of data and materials

The datasets supporting the conclusions of this article are included within the article.

### Ethics approval and consent to participate

Approval is not applicable as a case report is not regarded as a study according to the “Ethical Guidelines for Medical and Health Research Involving Human Subjects” of the Japanese Ministry of Health, Labour and Welfare.

### Consent for publication

Written informed consent was obtained from the patient for the publication of this case report, including her medical data and images.

### Competing interests

The authors declare that they have no competing interests.
